# A microfluidic array device for single cell capture and intracellular Ca^2+^ response analysis induced by dynamic biochemical stimulus

**DOI:** 10.1042/BSR20210719

**Published:** 2021-07-28

**Authors:** Wenbo Wei, Miao Zhang, Zhongyuan Xu, Weifeng Li, Lixin Cheng, Hongbao Cao, Min Ma, Zongzheng Chen

**Affiliations:** 1 First Affiliated Hospital of Shenzhen University (Shenzhen Second People’s Hospital), Shenzhen 518035, China; 2 Integrated Chinese and Western Medicine Postdoctoral Research Station, Jinan University, Guangzhou 510632, China; 3 Research Center for Clinical Pharmacology, Southern Medical University, Guangzhou 510515, China; 4 Department of Critical Care Medicine, Shenzhen People’s Hospital, The Second Clinical Medicine College of Jinan University, Shenzhen 518020, China; 5 School of Systems Biology, George Mason University (GMU), Fairfax, VA 22030, U.S.A.

**Keywords:** dynamic biochemical signal transmission, dynamic biochemical stimulation, intracellular Ca2+ dynamics response, microfluidic array, single cell capture

## Abstract

A microfluidic array was constructed for trapping single cell and loading identical dynamic biochemical stimulation for gain a better understanding of Ca^2+^ signaling at single cell resolution in the present study. This microfluidic array consists of multiple radially aligned flow channels with equal intersection angles, which was designed by a combination of stagnation point flow and physical barrier. Numerical simulation results and trajectory analysis have shown the effectiveness of this single cell trapping device. Fluorescent experiment results demonstrated the effects of flow rate and frequency of dynamic stimulus on the profiles of biochemical concentration which exposed on captured cells. In this microarray, the captured single cells in each trapping channels were able to receive identical extracellular dynamic biochemical stimuli which being transmitted from the entrance in the middle of the microfluidic array. Besides, after loading dynamic Adenosine Triphosphate (ATP) stimulation on captured cells by this device, consistent average intracellular Ca^2+^ dynamics phase and cellular heterogeneity were observed in captured single K562 cells. Furthermore, this device is able to be used for investigating cellular respond on single cell resolution to temporally varying environments by modulating the stimulation signal in terms of concentration, pattern, and duration of exposure.

## Introduction

With cells continually exposed to temporal varying biochemical microenvironment *in vivo*, the cytoplasmic Ca^2+^ transient dynamics are motivated and respond to external biochemical stimulus. The amplitude and duration of intracellular Ca^2+^ oscillations contribute to the differential activation of various transcription factors [1,2], leading to the different gene expression associated with cell differentiation, spreading, proliferation, migration and apoptosis, respectively [3,4]. However, the question remains unclear that how cells use this secondary messenger to elicit an extensive range of amplitude frequency-dependent responses. Because Ca^2+^ signaling events occur across a wide spatiotemporal range and in the context of a complex feedback network, analysis of Ca^2+^ regulation demands fine control of biological conditions and measurement of cellular responses.

In common method for cell stimulus-response analysis, cell population is considered as research object, and experimental results of this approach are transient average response of cell population [5,6]. However, this method cannot reflect the response of individual cells clearly. There are growing evidences showing that the individual single cells which even have same genetic material, are showing different reactions under same external stimuli [7]. For example, such mice pluripotent progenitor cells or cancer cell line contribute to different fates due to the heterogeneity of regulating protein [8], basic signal protein expression [9], or dynamics of regulating apoptosis protein [10]. However, these individual single cellular responsive behaviors are overshadowed by the average response of population cells. Therefore, it is extremely important to analyze the cellular heterogeneity in response to uniform stimulus in single cells. Current technological approaches limit the delivery of environmental cues and subsequent analysis of single-cell behavior for testing these theories.

As a powerful high-integrated experimental platform, microfluidics was used to simulate the microenvironment of cells or organs [11–13], especially offering the combined advantages of precision fluid control necessary for manipulation of single cells and exchange of concentration conditions around cells for parallel analysis dynamic stimulus-response in a single device [12,13]. By using microfluidic platform, single yeast cells trapped via partially closed valves in response to short repeated pulses of α-factor [14], single Jurkat T cells in response to low frequency H_2_O_2_ [15,16] and oscillatory Ca^2+^ stimulus [17] in microstructure well-trapping chip. Alternatively, single adherent cells cultured on the bottom of the microfluidic channel were also investigated for their responses induced by temporal varying stimulus, such as adenosine triphosphate (ATP) pulse stimulus on NIH-3T3 cells [18], HeLa cells [19] and human umbilical vein vessel endothelial cells (HUVECs) [20], as well as a brief bacterial lipase pulses on single macrophage cell [21]. Nevertheless, there are two issues to be considered carefully in the study of dynamic external stimuli-induced cell responses in microfluidic channels. First, it has been proved that microfluidic channel is a low-pass filter [22,23], signal attenuation will be occurring in transmission of dynamic stimulating signal in microfluidic channel. It easily gives rise to deliver different stimuli on individual cells at different locations. Especially in this type of trapping well channels, which were designed as long channels on account of capturing more single cells, therefore, the results in the dynamic stimulation experienced on each cells at different locations in microchannel were diverse, which contribute to increase in the complexity of the single-cell resolution analysis of dynamic stimuli-response in single cell. Second, loading dynamic signals on cells requires controlling flow which will induce stimulus of shear stress on cells which were cultured or captured in microchannel generally, which leads to affecting the results of dynamic stimulus-response studies on cell.

**Figure 1:**
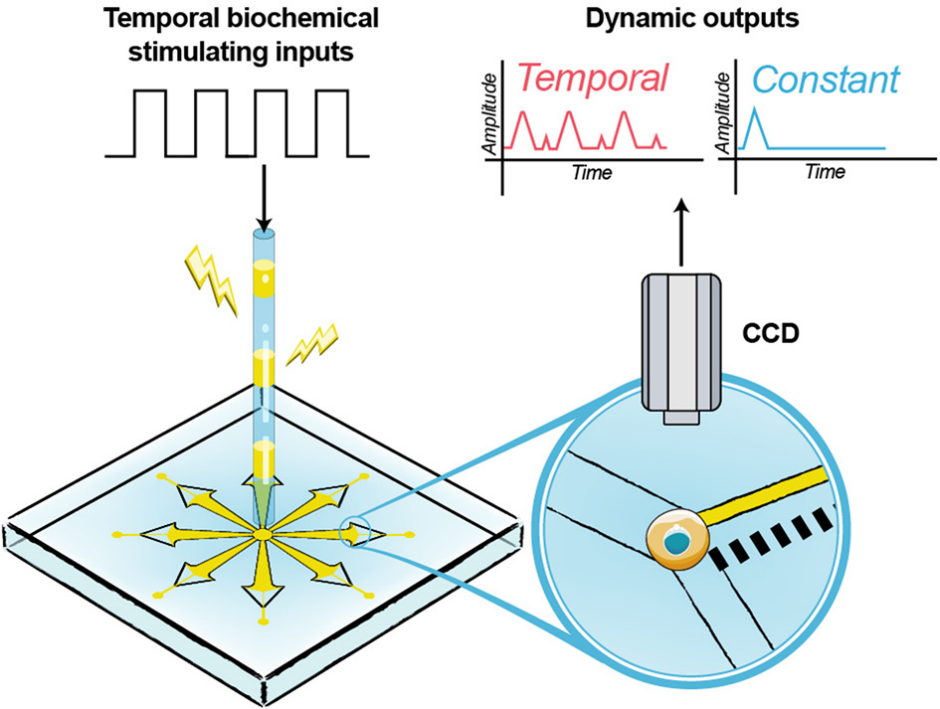
Design of the microfluidic array device After single cell capture, the dynamic biochemical stimuli were loaded from the entrance at the middle of the microfluidic array, then the intracellular Ca^2+^ dynamics of single cell were observed and analyzed by an inverted microscope with a CCD camera.

To analyze the intracellular Ca^2+^ dynamic responses to identical dynamic biochemical stimulations in single cells without influence of flow-induced shear stress, a microfluidic array device (Figure 1) is proposed by integrating eight equiangular radial trapping units which was designed by the principle of combined stagnation point flow and physical barrier as before [24]. This microfluidic array device provides the captured single cells experiencing coincident stimuli of dynamic biochemical signal from the entrance at the middle of the microfluidic array. The effects of temporal ATP stimulation on the intracellular Ca^2+^ dynamics in single K562 cells are investigated by using the proposed microfluidic array.

## Materials and methods

### Design of the single cell trapping microchannel

**Figure 2:**
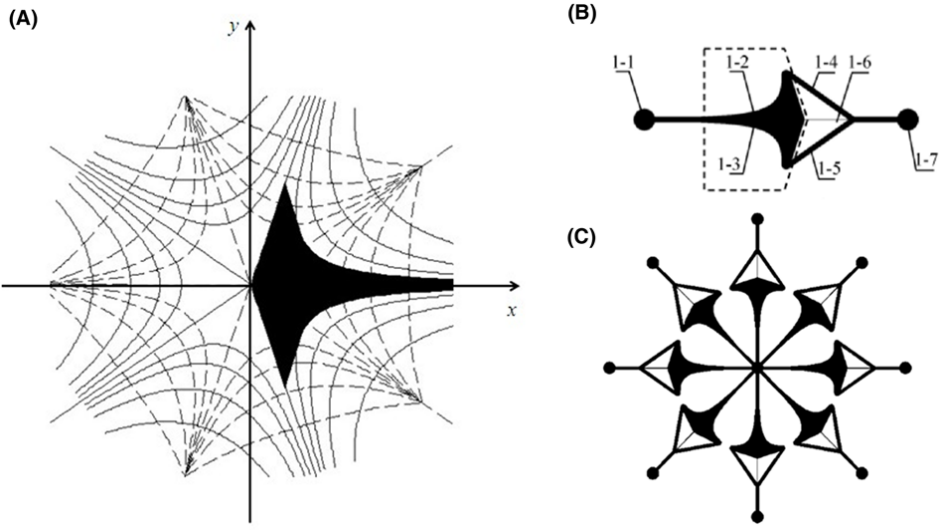
Principle and structure schematics of the single cell capturing microfluidic array (**A**) The shallow microfluidic channel in the flow field when *n* is 2.5. (**B**) Single cell trapping microchannel unit, 1-1 medium inlet; 1-2, 1-3 curved boundaries; 1-4, 1-5 outlet channels; 1-6 resistance channel; 1-7 fluid outlet; (**C**) 8-units microfluidic array.

Utilizing the complex potential function *W* = *AZ^n^*, where *A* is a real number and *n* is a positive number greater than 1 [24], the microchannel with fluid stagnation point was constructed as shown in Figure 2A,B [24]. For trapping enough single cells and loading identical dynamic biochemical stimuli with each captured single cell, several trapping microchannel units are arranged in an equiangular radial array. Based on this, an 8-unit single cell trapping microfluidic array is exhibited in Figure 2C.

### PDMS-glass microfluidic chip fabrication

All the microchannels were patterned in PDMS (Sylgard 184, Dow Corning, Midland, MI, U.S.A.) by replica molding. The mold was prepared by spin coating a thin layer of negative photoresist (SU-8, MicroChem, Westborough, MA, U.S.A.) on to a single side polishing silicon wafers and patterned with ultraviolet (UV) exposure. Next, the microchannel layer was obtained by pouring PDMS with 10:1 (w/w) base:cross-linker ratio on to the mold yielding a thickness of 3 mm roughly. After curing the elastomer for 2 h at 80°C, the PDMS slab was peeled from the mold, punched, and hermetically bonded to a coverslip by plasma oxidation.

### Implementation of integration system

**Figure 3:**
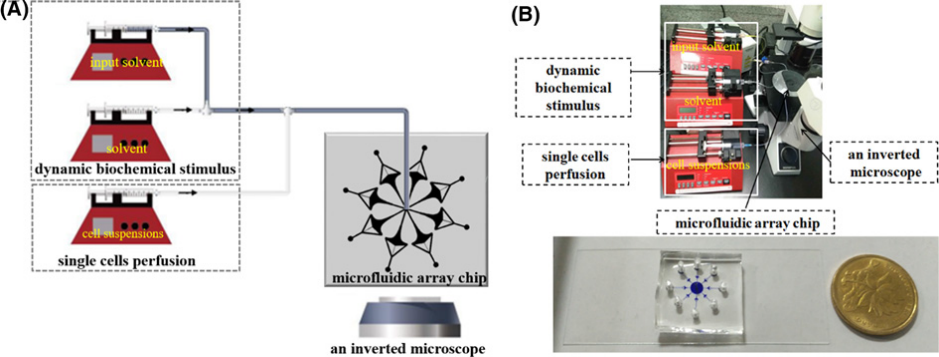
Single cell capture microfluidic array and its dynamic biochemical stimulus drive system The schematics (**A**) and actual diagram (**B**) of the 8-unit microfluidic array and integrated system. The system mainly incorporates a dynamic biochemical stimulus module which implement time-varying biochemical concentration by controlling the flow rates of input solvent and solvent over time; a single cell perfusion module, an 8-unit microfluidic array chip, and an inverted microscope for observing real-time changes of intracellular Ca^2+^ signal.

After the PDMS-glass microfluidic array chip was fabricated as shown in Figure 3B, it was connected with three syringe pumps (NE-1000, New Era, U.S.A.) for controlling the dynamic biochemical stimulation and single cell suspension perfusion by regulating the flow rates from the three syringe pumps and T-bend. More specifically, as shown in Figure 3A, the entrance of microfluidic array was connected to one syringe pump for perfusion of single cell suspension and two syringe pumps for loading dynamic biochemical stimulation which coupling by a T-bend and silicone tubes. These dynamic biochemical stimuli were generated by controlling the flow rates of the input solution and the solvent from two syringe pumps, respectively [25]. An inverted microscope (CKX41, OLYMPUS, Japan) equipped with a CCD camera (DS126431, CANON, Japan) was adopted to observe the profiles of dynamic biochemical stimuli and the intracellular Ca^2+^ signal in single cells which were captured at the stagnation point of the microchannel in real time. The actual experimental system was finally set up as shown in Figure 3B.

### Numerical simulation of the height-averaging velocity in microfluidic channel

The numerical simulations for velocity field and wall shear stress were conducted using computational fluid dynamics (CFD) package ANSYS 15.0 (Ansys Inc). Briefly, the cell trapping microchannel was divided into the hexahedral and tetrahedral grids (135564 nodes, 120720 elements) in the Workbench Mesh, full Navier–Stokes equations under steady flow were solved in FLUENT 6.3. The geometrical and fluid parameters used in all the simulations are given in Table 1.

**Table 1:** Default values of geometrical size and fundamental fluid parameters used in numerical simulations

Parametric description	Symbol	Value (unit)
Length of cell trapping microchannel (*x*-direction)	*L*	0.1 cm
Entrance width of cell trapping microchannel (*y*-direction)	*W*	50 µm
Height of microfluidic channel (*z*-direction)	*H*	80 µm
Parameter of the complex potential function	*n*	2.5
Fluid viscosity	*η*	0.001 Pa.s

### Cell capturing trajectory and velocity analysis

K562 cells were obtained from American Type Culture Collection (ATCC), cultured in Dulbecco’s modified Eagle’s medium (DMEM) supplemented with 10% fetal bovine serum (FBS), 2 mM l-glutamine, 1 unit.mL^−1^ penicillin, and 100 mg.L^−1^ streptomycin (Invitrogen, U.S.A.), and were maintained at 37°C with 5% CO_2_ in culture flask.

Before simple particle (single K562 cells) tracking experiment was performed, K562 cells (1 × 10^6^ cells/mL) were resuspended in 1 ml of fresh DMEM, then the single cell suspensions were divided into microchannel by syringe pump from the center entrance of microfluidic array chip. The trajectories of the K562 cells were recorded by and the microscope with a CCD camera. The velocity of K562 cells were calculated and analyzed by the K562 cells trajectories along the central axis *x*-direction.

### Experimental validation of dynamic biochemical stimulations

For easy detection, the dynamic biochemical stimulations in the microfluidic array device were experimentally validated using the fluorescent solution (Rhodamine-6 Sigma) instead of dynamic biochemical stimuli itself. The concentration of fluorescent solution changing as a square-wave-like signal with frequency at 1/3, 1/6, 1/12, 1/30, and 1/60 Hz was input through the entrance at the center of the microfluidic array device at volumetric flow rate (*Q*) at 5.4, 3.6, and 1.8 mL/h, respectively. The time-varying images for dynamic fluorescent profiles at 0.05 and 0.1 mm (near stagnation point) along *x*-direction in cell capturing microchannel units were observed and detected by the fluorescence microscope with a CCD camera (Figure 3B). The dynamic fluorescent intensities were then extracted from the images using MATLAB (The Math Works R2009b, Inc). While the fluorescent intensities at each time point were calculated, the gray values from the image background were subtracted. All the experimental results were normalized to a constant reference value.

### Intracellular calcium dynamic response analysis

K562 cells were resuspended in 1 ml of fresh DMEM with Fluo3-AM (Sigma, 5 mM, final concentration) without FBS. After 1 h of incubation at room temperature, cells were re-suspended in Hanks’ balanced salt solution (HBSS) buffer and incubated at 37°C in the 5% CO_2_ environment for 30 min. Then cells were centrifuged and re-suspended at 10^6^ cells.ml^−1^ in HBSS buffer ready for on-chip analysis. Afterwards, the chip was flushed and refilled with DMEM supplemented with 10% FBS. When signal cell was captured in microfluidic array, the syringe pumps would start up according to the designed program as dynamic biochemical stimulations that described in the previous to generate a dynamic ATP (Sigma, U.S.A.) stimulation. The time-varying fluorescent images for the intracellular Ca^2+^ response in K562 cells at the regions of interest were recorded in a sampling frequency (one frame per 3 s) with the CCD for 4 min at room temperature. The Ca^2+^ dynamic fluorescent intensities were then extracted from the dynamic images using the same method as described for dynamic fluorescent images in previous subsection 2.6.

## Results

### Numerical simulation of the height-averaging velocity in trapping microfluidic channel unit

**Figure 4:**
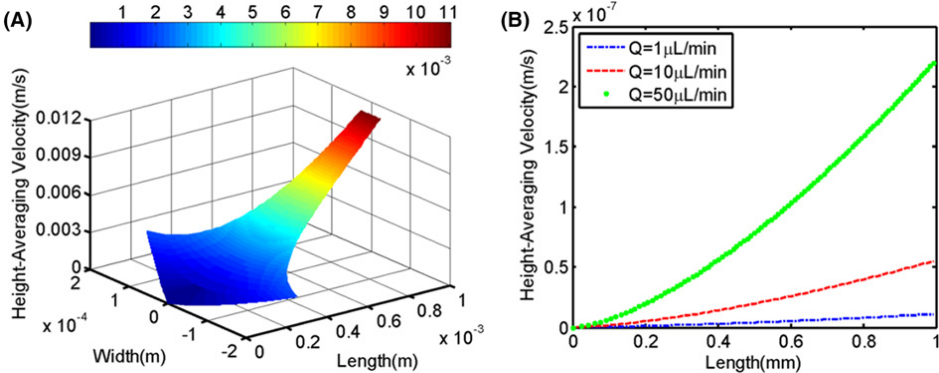
Numerical simulation of the height-averaging velocity in a single cell capture channel unit (**A**) The height-averaging velocity profile in 3-D of the flow chamber when *n* is 2.5. (**B**) The height-averaging velocity in the chamber along the *x*-axis with different flow rate. The blue, red, and green lines represent the results when the inlet flow rate *Q* is 1, 10, and 50 μl/min, respectively.

To verify the availability of the single cell capture channel that we designed, average flow velocity of single cell capture channel unit was explored by numerical simulation. When the resistant channel (in Figure 3A) was completely blocked, the velocity in capture channel decreases gradually from maximum to zero along the central axis as shown in Figure 4A, demonstrating the existence of stagnation point flows at the cross-point. Even at different flow rates, 1, 10, and 50 μL/min, respectively, the height-averaging velocity along the *x*-axis also tends to go from maximum to zero (Figure 4B). It can be seen that the designed microfluidic channel conforms with the characteristic of stagnation point flow, theoretically, as single cell approaches the stagnation point, the velocity of cell is zero, that is, cell was captured. Additionally, due to the spatial limitation of physical barrier, the target can be captured stably.

### Trajectory and velocity analysis in the capturing process of single cell

**Figure 5:**
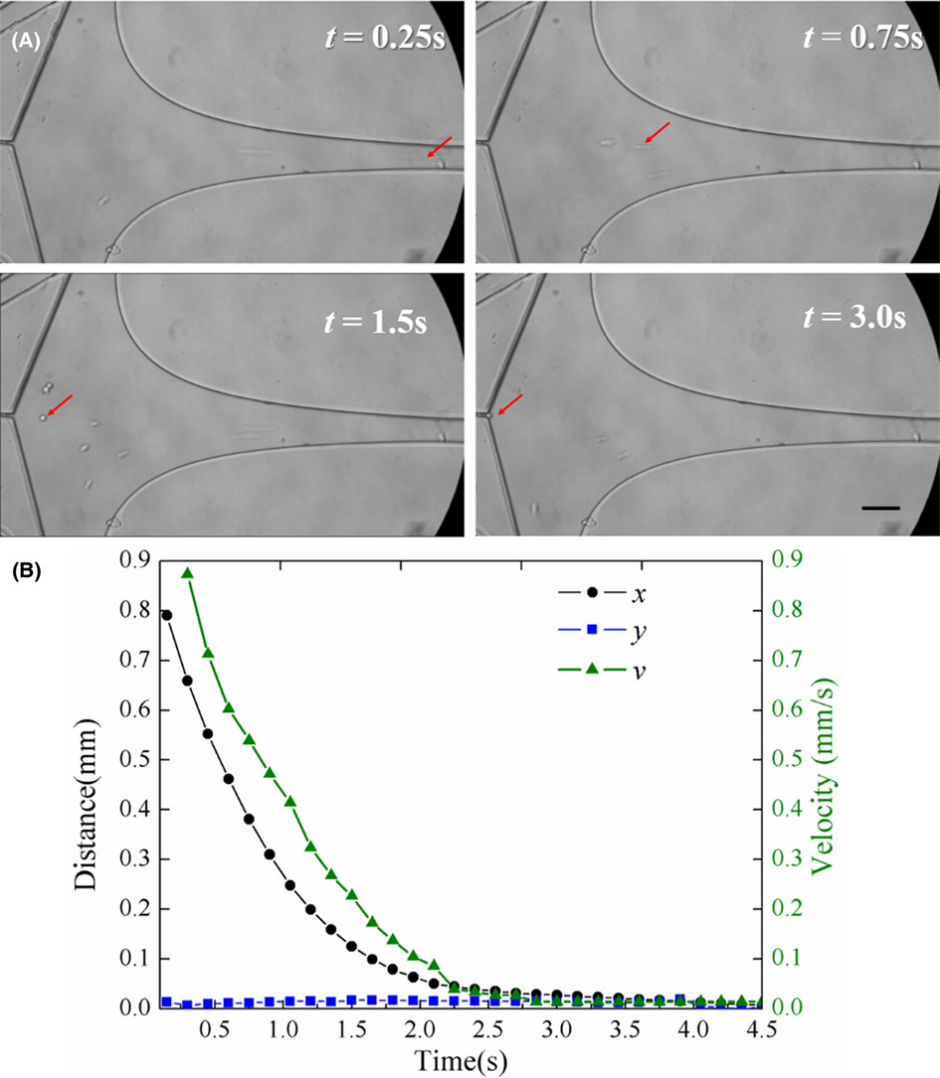
Trajectory and velocity analysis in a single cell trapping process (**A**) A single K562 cell in the trapping microchannel at different time. (**B**) The distance along *x*-direction and *y*-direction and the velocity along central axis *x*-direction of a trapping cell from the entrance to the trapping point. Scale bar is 50 μm.

The proof-of-principle experiment of the stagnation point flow trapping were performed by analyzing the trajectory and velocity of single cell from the entrance to the trapping point in cell capturing unit. In Figure 5A, the single K562 cell in trapping process was marked with a red arrow, the cell that will be captured was at the entrance of the trapping channel (*t* = 0.25 s), then moved in the front of the trapping channel (*t* = 0.75 s). The cell kept moving towards the trapping point in the following (*t* = 1.5 s). Finally, the cell was captured at the trapping point (*t* = 3.0 s). In more details, the trajectories of cell capturing are shown in Figure 5B, in this process, the *y*-direction distance of cell essentially unchanged, it is suggested that the cell moves along the central axis of trapping channel. Besides, the *x*-direction distance and moving velocity of cell from the entrance to trapping point decreased rapidly at first, then as approaching the stagnation point, the velocity of the single cell decreased gradually, until the location of the stagnation point where the value became zero. Taken together, these results are consistent with what we hypothesizing about the device designing.

### Experimental verification of dynamic biochemical stimulation loading

In our platform, dynamic biochemical stimulation was delivered in forms of temporal varying boluses which generated by controlling the flow rates of the input solution and the solvent. In order to make clear that the actual time-varying biochemical stimuli exposed on each captured single cell in our microfluidic array chip, it is described that how transport of these signals can be affected by dispersion and convection in the variable cross-section microfluidic channel. The chemical stimulus profiles at various flow rates (*Q*) and frequency of input stimulus (*f*_C_) were empirically characterized.

**Figure 6:**
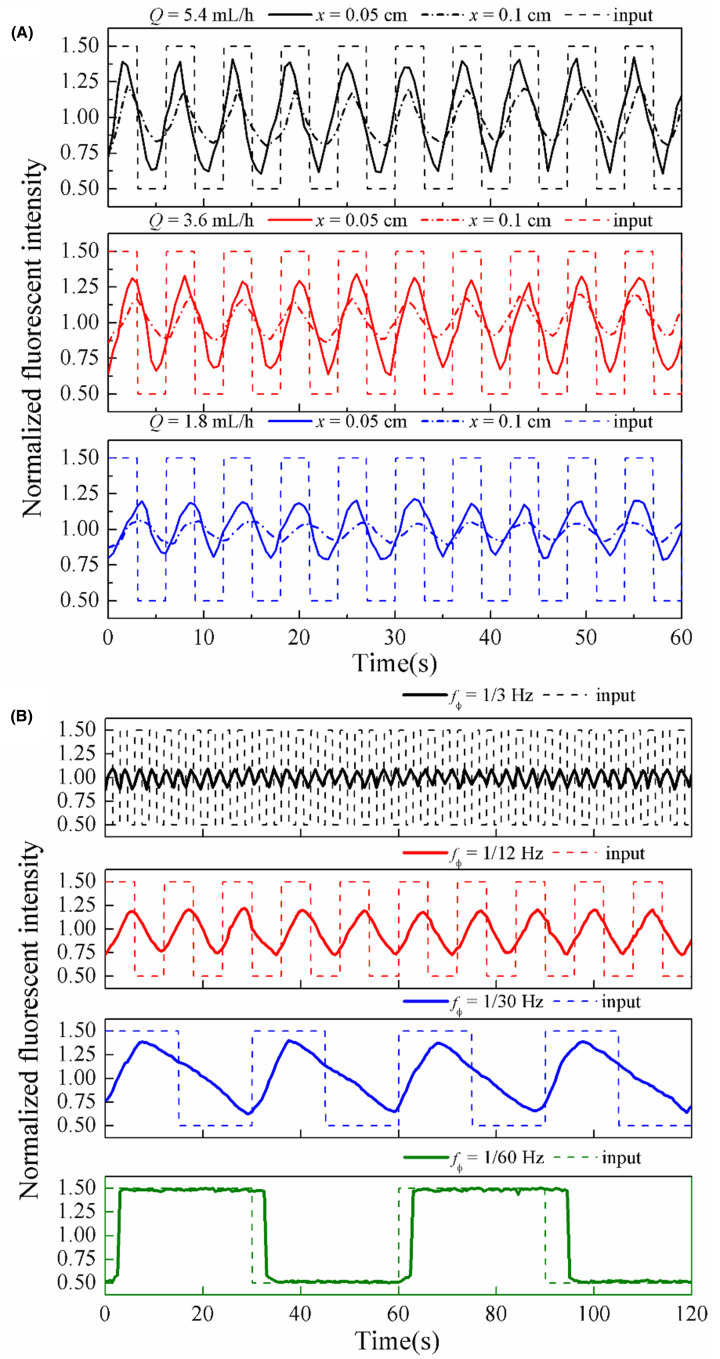
Stimulus profiles in cell capturing microchannel with input dynamic stimuli with different flow rates and signal frequency, *f_C_* (**A**) Transmission of square wave fluorescence stimuli signals at *f_C_* = 1/6 Hz in the steady flow at *Q* = 5.4, 3.6, and 1.8 mL/h, respectively. Experimental results detected at *x* = 0.05 and 0.1 cm. (**B**) Transmission of square wave fluorescence signals at *f_C_* = 1/3, 1/12, 1/30, and 1/60 Hz in the steady flow at *Q* = 5.4 mL/h. Experimental results detected at *x* = 0.1 cm.

The effect of flow rates on stimulus profiles was examined firstly. Figure 6A shows the resulting temporal profiles of fluorescent intensity of fluorescent solution driven with flow rates at 5.4, 3.6, and 1.8 mL/h, respectively. It is can be seen that increased flow velocities under large flow rates result in less attenuation throughout the microfluidic channel. Additionally, as the transmission distance *x* increases, attenuation effect is more clearly observed. Near the stagnation point (*x* = 0.1 mm), the fluorescence intensity is lower than at the middle of the variable cross-section microfluidic channel (*x* = 0.5 mm).

Then, the effect of *f*_C_ on stimulus profiles was studied. Figure 6B shows the transmission of square wave fluorescence signals at *f*_C_ = 1/3, 1/12, 1/30, and 1/60 Hz in the steady flow with the flow rate *Q* = 5.4 mL/h. At low frequency (*f*_C_ = 1/60 Hz), the detected profile well preserves the stimulus profile introduced from the inlet. As *f*_C_ increases, the detected profile amplitude reduces. Moreover, the high frequency components indicated by the sharp change in waveform are filtered out, leading to the change in waveform from a square wave to a triangle-like wave and eventually to a sine-like wave. These phenomena reveal that a filtering effect acts on time-varying fluorescence stimuli through the transmission in steady flows. The filtering effect significantly enhances with the increase in signal frequency *f*_C_ (Figure 6B).

Additionally, one goal of our device is to interrogate each captured cells with identical stimulus signals spanning broad timescales. Under inputting *f*_C_ at 1/30 Hz, the stimulus profiles of each trapping point were inspected as shown in Supplementary Figure S1. It is can be seen that the single or average dynamic stimulus exposed on each cell capture point was almost identical.

### The response of intracellular Ca^2+^ concentration under temporal ATP stimulus in single K562 cell

**Figure 7:**
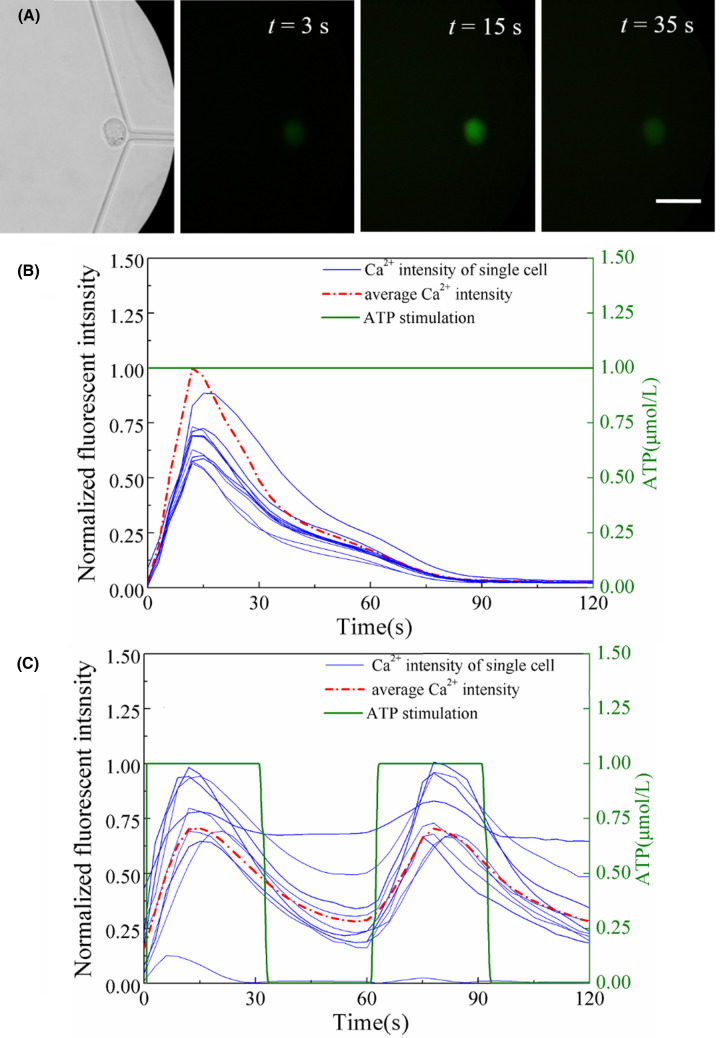
The intracellular Ca^2+^ dynamics of single K562 cell in response to constant and dynamic ATP stimulations (**A**) The intracellular Ca^2+^ intensity of single K562 cell captured at the stagnation point at *t* = 3, 15, and 35 s, respectively. Scale bar is 100 μm. (**B**) The intracellular Ca^2+^ dynamics in K562 cell in response to constant ATP stimulation with concentration at 1 μmol/l. (**C**) The intracellular Ca^2+^ dynamics in K562 cell in response to dynamic ATP stimulation with concentration is changing as asquare wave with a period of 60 s between 1 and 0 μmol/l. The average Ca^2+^ intensity is the average value of ten single cells from two individual experiment groups. All the data are normalized to a constant reference value.

In order to observe the intracellular Ca^2+^ dynamics in response to the dynamic ATP stimuli at single cell level, the single K562 cell captured at the stagnation point in microchannel and its dynamic responses of the intracellular Ca^2+^ concentration induced by static and dynamic ATP stimulations were carefully measured in the device were shown in Figure 7A. As a specific case, the intracellular Ca^2+^ intensity in the single K562 cell at 3, 15, and 45 s were exhibited respectively under the stimulation of constant ATP stimulation with concentration at 1 μmol/L.

It can be seen that in Figure 7B after motivated by this constant ATP stimulation, as increases in time, the intracellular Ca^2+^ concentration increases to a maximum and then deceases to the original value, only single peak Ca^2+^ response is observed for this case. However, once with a dynamic ATP stimulation which concentration is changing as a square wave with a period of 60 s between 1 and 0 μmol/l, a second dynamic response of intracellular Ca^2+^ concentration with the same frequency as that of ATP stimulation can be observed shown in Figure 7C. It is easy to noticed that the changing of intracellular Ca^2+^ concentration in single cells are heterogeneous even under identical constant and dynamic stimulations from select individual cell traces are shown in Figure 7B,C. These results demonstrate that this device to generate biologically relevant signals in order to interrogate cellular signaling transduction respond to temporally varying environments.

## Discussion

As a powerful single-cell integrated experimental platform, lots of researchers have been to capturing single cells at a specific location in microfluidics by used control valves [26], physical barrier array [27], hydrodynamics [28], non-intrusive optical capture [29], surface chemical modification [30] etc. The principle of capturing single cells in this study was combined stagnation point flow and physical barrier as previously [24]. This system can capture enough cells in a hydrodynamic manner, and physical barriers could ensure the stability of the captured cells which were performed with further stimulation. From the results of numerical simulation of the velocity field and single particle tracing shown in Figures 3 and 4, respectively, the velocity at the trapping point was keeping at zero with flow from inlet with different flow rates. In Johnson et al.’s study [28], the cells were captured by principle of stagnation point flow need to keeping external flow condition stabilization, this is not convenient to analyze response of trapped cells to external concentration varying stimulation. In this study, the cells were captured at stagnation point for enough time to ensure next stimulus–response experiment through a design of combined physical barrier with stagnation point flow. Besides, to avoid the effect of shear stress additional porous membrane was integrating between cell layer and stimulus flow layer [31], whereas additional membrane increases the complexity of device construction and the biochemical signal transmission to cells.

It is noteworthy that the microchannels acts as a low-pass filter are dependent on multiple factors including the biochemical signal frequency, flow rates and signal transporting distance in our previous study [20,22]. Therefore, the biochemical stimuli exposed on cells may be different from expected. To assess the performance of load biochemical stimuli on captured single cells in microfluidic array precisely, the transmission characteristics of dynamic biochemical stimulations are investigated through numerical simulation and fluorescence solution experiment. Figure 6A indicated that the lower flow rate and longer transporting distance will give rise to stronger signal attenuation induced by rise time in the microchannel. As frequency of stimulus signal was becoming lower, the signal attenuation was weakening, as shown in Figure 6B, until the frequency at 1/60 Hz, the attenuation of stimulus hardly appeared. For a better signal loading on cells in the microchannel, signal filtering and its influence factors should be carefully taken into. For example, in studies on delivering stimulus for trapping of single cells by micro wells, the microfluidic construction was designed with long microchannel for increasing the numbers of trapping unit [15,16]. This results pointed that the dynamic stimulation experienced on each cells at different location in microchannel were not the same.

In the present study, to ensure each captured cell received coincident dynamic stimulation and capture enough cells, the design of equal structure units was arranged in forms of equal radial angle which guarantee the distance from dynamic stimulus entrance to cell trapping point are exactly identical (Supplementary Figure S1).

The responses of consistent genetic derived single cells are exhibited diversity under identical stimuli due to cellular heterogeneity [5–10]. This study will help to avoid cell population response overshadowed single cellular behaviors on account of heterogeneity. It is shown that in Figure 7B,C, the cellular heterogeneity about Ca^2+^ response in single K562 cells was demonstrate under uniform dynamic stimulus. Besides, temporal response analysis will extract more of this encoded information and shed light on the relationship between external stimulus and intracellular Ca^2+^ dynamics. The cytoplasmic Ca^2+^ transient dynamics (frequency, amplitude, and duration of oscillation) was sensitive to the biochemical changing in extracellular environment, which have been commonly regarded as a critical factor in regulating cellular function and behavior through dynamic variation of transcription factors [1–4]. By this device, the cytoplasmic Ca^2+^ transient was observed in single K562 cell which exposed to a square-wave-like ATP stimulus with flow rate and frequency at 5.4 mL/h and 1/60 Hz, respectively. The results suggest that a second dynamic response of intracellular Ca^2+^ concentration with the same period as frequency of ATP stimulus. By modulating the temporal signal in terms of concentration, pattern and duration of exposure, we can study how cells respond to temporally varying environments. This single cell study platform, in combination with live cell imaging and dynamic stimulation generator, may be useful in elucidating signal transduction pathways present on multiple time scales and cellular heterogeneity in the future.

## Conclusion

A microfluidic device for analyzing single cells in response to dynamic cues of microenvironment was constructed by a cell capture microfluidic array which based on combined stagnation point flow with physical barrier. By connecting a dynamic biochemical stimulus generator at the center entrance of the array chip and fluorescent solution, the factors resulting in the attenuation of dynamic stimuli in this variable cross-section was analyzed carefully. Cellular Ca^2+^ dynamics of single K562 cells were observed under a precise dynamic biochemical stimulus which considered sufficiently these avoiding attenuation factors. Preliminary experimental results of average intracellular Ca^2+^ dynamics of single cells show that the same period as low frequency ATP stimulus.

## Supplementary Material

Supplementary Figure S1

## Data Availability

All supporting data are included within the main article and its supplementary files.

## Abbreviations

ATP: adenosine triphosphate
DMEM: Dulbecco’s modified Eagle’s medium
FBS: fetal bovine serum
HBSS: Hanks’ balanced salt solution
HUVECs: human umbilical vein vessel endothelial cells
