# Supplementary material for: A microfluidic array device for single cell capture and intracellular Ca2+ response analysis induced by dynamic biochemical stimulus
Source: Biosci Rep. 2021 Jul 28;41(7):BSR20210719. doi: 10.1042/BSR20210719 (PMC8319492; doi:10.1042/BSR20210719)
Supplement: Supplementary Figure S1 [file BSR-2021-0719_supp.pdf]

Supplementary Information

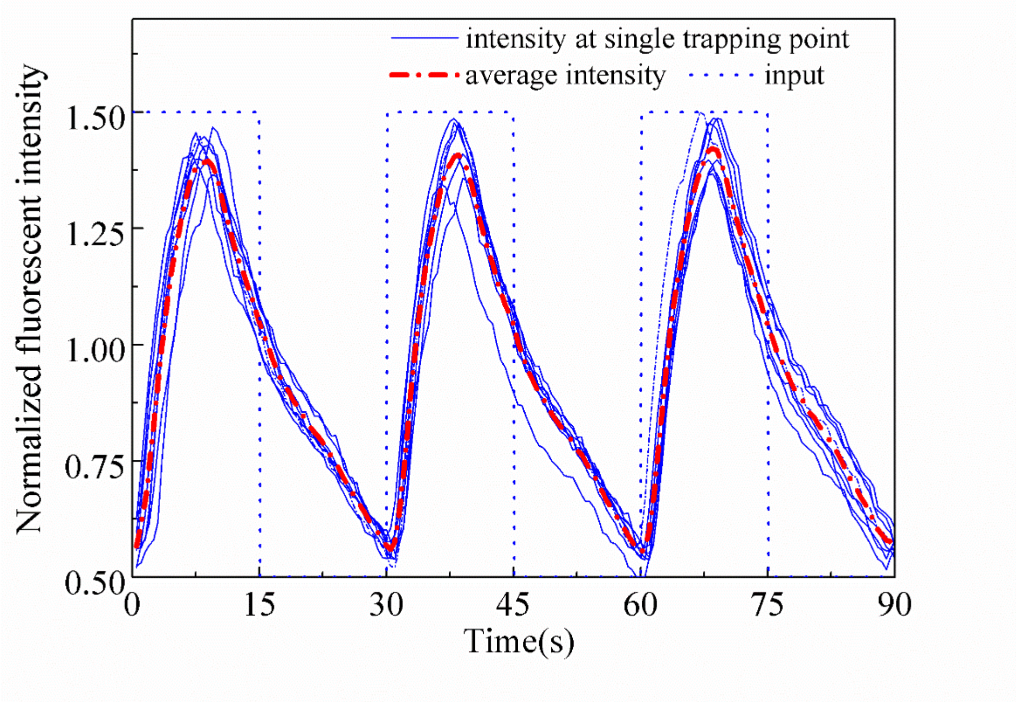

Figure S1. Individual and average fluorescent stimulus profiles at individual cell capturing points of microfluidic array.

Stimulus profiles at cell capturing point of microfluidic array with input dynamic stimuli with frequency f_C = 1/60 Hz in the steady flow at Q = 5.4 mL/h. Experimental results detected at x = 0.1 cm.
